# An Untargeted Lipidomics Workflow Incorporating High-Resolution Demultiplexing (HRdm) Drift Tube Ion Mobility-Mass Spectrometry

**DOI:** 10.1021/jasms.4c00251

**Published:** 2024-09-14

**Authors:** David C. Koomen, Jody C. May, Alexander J. Mansueto, Todd R. Graham, John A. McLean

**Affiliations:** † Center for Innovative Technology, Department of Chemistry, Vanderbilt University, Nashville, Tennessee 37235, United States; ‡ Department of Biological Sciences, Vanderbilt University, Nashville, Tennessee 37235, United States

**Keywords:** untargeted lipidomics, HRIM, peak deconvolution, Hadamard transform, murine dyslipidemia

## Abstract

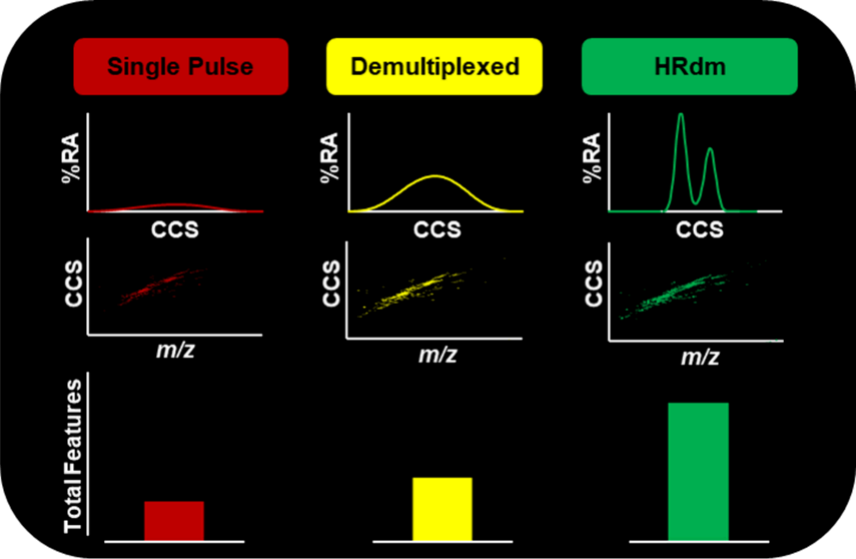

Global discovery lipidomics can provide comprehensive chemical information toward understanding the intricacies of metabolic lipid disorders such as dyslipidemia; however, the isomeric complexity of lipid species remains an analytical challenge. Orthogonal separation strategies, such as ion mobility (IM), can be inserted into liquid chromatography-mass spectrometry (LC-MS) untargeted lipidomic workflows for additional isomer separation and high-confidence annotation, and the emergence of high-resolution ion mobility (HRIM) strategies provides marked improvements to the resolving power (*R*_p_ > 200) that can differentiate small structural differences characteristic of isomers. One such HRIM strategy, high-resolution demultiplexing (HRdm), utilizes multiplexed drift tube ion mobility spectrometry (DTIMS) with post-acquisition algorithmic deconvolution to access high IM resolutions while retaining the measurement precision inherent to the drift tube technique; however, HRdm has yet to be utilized in untargeted studies. In this manuscript, a proof-of-concept study using ATP10D dysfunctional murine models was investigated to demonstrate the utility of HRdm-incorporated untargeted lipidomic analysis pipelines. Total lipid features were found to increase by 2.5-fold with HRdm compared to demultiplexed DTIMS as a consequence of more isomeric lipids being resolved. An example lipid, PC 36:5, was found to be significantly higher in dysfunctional ATP10D mice with two resolved peaks observed by HRdm that were absent in both the functional ATP10D mice and the standard demultiplexed DTIMS acquisition mode. The benefits of utilizing HRdm for discerning isomeric lipids in untargeted workflows have the potential to enhance our analytical understanding of lipids related to disease complexity and biologically relevant studies.

## Introduction

Traditional molecular discovery in mass spectrometry (MS)-based untargeted lipidomics has been hindered by the large number of isomeric compounds inherent to lipid species.^1,2^ Contemporary lipidomic workflows utilizing hyphenated MS strategies, such as liquid chromatography-tandem mass spectrometry (LC-MS/MS), provide high-confidence molecular annotation on small sample volumes with relatively high throughput. These techniques are now well supported by computational pipelines; however, isomeric identification and annotation is still challenging.^3−7^ Integrating ion mobility (IM) analysis into these lipidomic workflows can provide additional separation of isomeric lipid features; however, another data layer such as IM complicates the computational requirements for processing and analyzing the data.^8−14^ Efforts to simplify untargeted IM data processing and analysis are ongoing, although no unified data processing strategy currently exists.^10,15−20^

Drift tube ion mobility spectrometry (DTIMS) is the original implementation of the IM technique where separation is accomplished by a uniform electric field in an inert buffer gas (typically helium or nitrogen) yielding distinct arrival time distributions (ATDs) from which highly-reproducible collision cross section (CCS) values can be extracted using a fundamental relationship.^21,22^ As such, DTIMS is considered the most accurate means of obtaining the gas-phase CCS and DT measurements, which are routinely used to calibrate measurements from other IM techniques. The arrival time measurements from DTIMS experiments are traditionally obtained using a single-pulse gate release sequence, where ions are trapped and accumulated for a period of time (e.g., milliseconds) and then released into the drift tube for mobility separation and subsequent detection. To address the relatively low sensitivity and throughput of this conventional pulse-and-wait experiment, time multiplexing strategies have been developed which introduce multiple ion pulses per measurement cycle that are subsequently deconvoluted using an inverse transformation of the known pulse sequence.^23−28^ Multiplexed IM can increase sensitivity by nearly an order of magnitude compared to conventional single pulse IM; however, multiplexing does not significantly improve the resolution of the IM separation, which for commercially available DTIMS the resolving power (*R*_p_) benchmarks are in the range of 50–60.^29,30^ To address resolution limitations, a post-acquisition data processing strategy referred to as high-resolution demultiplexing (HRdm) has been developed, which provides an IM resolution improvement of up to four times what is observed in the unprocessed data (*R*_p_ ≈ 50 vs >200) while retaining the high CCS accuracy and full broadband spectral outputs inherent to DTIMS. Despite these desirable figures of merit, HRdm has thus far been utilized exclusively for targeted analysis, and deployment into untargeted workflows has yet to be demonstrated.^30−37^ Here, a proof-of-concept study using *Atp10D*^*+/+*^ and *Atp10D*^*–/–*^ murine plasma lipid extracts with LC-IM-MS supported by HRdm was used to demonstrate the capability of HRdm to be incorporated into and improve upon untargeted lipidomic analyses. HRdm operates at a sufficiently high resolving power (*R*_p_ > 200) to distinguish isomeric lipid species, informing a more accurate identification for their biological impact related to dyslipidemia. The inclusion of the high-resolution ion mobility (HRIM) capabilities of the HRdm post-processing strategy combined with LC and high-resolution MS measurements (LC-HRIM-HRMS) provides a net benefit to the total number of distinguishable lipid features for improved lipidome investigation.^38^

## Methods

### Chemical Materials

High-purity (Optima grade) water, methanol, acetonitrile, isopropyl alcohol, formic acid, and chloroform were purchased from Fisher Scientific. *tert*-Butyl methyl ether (MTBE) and ammonium formate were purchased from Sigma-Aldrich. SPLASH LIPIDOMIX heavy-labeled lipid standards mixture was obtained from Avanti Polar Lipids.

### Sample Preparation

All mouse experiments were approved by the Vanderbilt Institutional Animal Care and Use Committee (IACUC) for use by the Graham Lab. C57Bl/6J mice contain a naturally occurring nonsense mutation in the *Atp10D* locus.^39^ Site-directed mutagenesis was used to develop corrected (+/+, functional *ATP10D*) and original (−/–, dysfunctional *ATP10D*) C57Bl/6J mice by the Graham Lab and Vanderbilt Genome Editing Resource to investigate dyslipidemia related to ATP10D dysfunction.^40,41^ Mouse plasma was collected at the end point of a 12-week high-fat diet (60% kcal fat, D12492, Research Diets Inc.). Mice were euthanized following an IACUC-approved euthanasia method involving CO_2_ asphyxiation and a secondary method of cervical dislocation. Blood was collected post-mortem via cardiac puncture. Plasma was isolated using EDTA as an anticoagulant followed by centrifugation at 1000 RCF for 15 min at 4 °C. The supernatant (plasma) was collected avoiding the buffy coat and red blood cell pellet.^42^ C57BL/6J mice have a naturally occurring stop codon mutation in the *Atp10D* gene that disrupts its function.^42^ CRISPR/Cas9 mutagenesis was used to correct this mutation to produce a functional Atp10D (+/+, corrected *Atp10D*) for comparison to the original strain (−/–, dysfunctional *Atp10D*).^41^ Lipids from female mouse plasma were extracted using MTBE after protein precipitation and drying and then reconstituted in 90:10 methanol/chloroform.

### LC-MS/MS and LC-IM-MS Analysis

Samples were analyzed with both LC-MS/MS and LC-IM-MS utilizing online LC (1290 Infinity, Agilent Technologies) coupled to a drift tube ion mobility-mass spectrometer (6560 Ion Mobility-QTOF, Agilent) using positive and negative ion mode electrospray ionization (Jet Stream, Agilent). Mass calibration was performed prior to the analyses. LC-MS/MS analysis was conducted with the top-2 iterative data-dependent acquisition (DDA) using three consecutive sample injections. The IM stage was operated in both single pulse and 4-bit multiplexing modes using nitrogen drift gas. The following mobile phases were used for LC separation: (A) water with 10 mM ammonium formate and 0.1% formic acid and (B) 60:36:4 isopropyl alcohol/acetonitrile/water with 10 mM ammonium formate and 0.1% formic acid. A 30 min LC gradient was performed on a reversed-phase column (Hypersil Gold HPLC C18, 1.9 μm particle size, 2.1 mm × 100 mm, 25002-102130, Thermo Scientific). Details for the LC method can be found in Table S1. Details on source conditions, instrument settings, and data acquisition parameters for single pulse and 4-bit multiplexing can be found in Tables S2 and S3, respectively.

### Data Processing

This workflow was performed on a 13th Gen Intel(R) Core i7-13700 2.10 GHz processor with 32 GB of RAM and 24 logical processor cores. The total time required to process a single multiplexed data file to a high-resolution demultiplexed file is around 4–5 h per multiplexed LC-IM-MS file. Additionally, data storage is increased by 8–10-fold using the workflow outlined here for a 30 min LC separation. Single pulse and 4-bit multiplexed ion mobility files were IM calibrated using a series of hexakis(fluoroalkoxy)phosphazene calibrants (HFAPs, ESI-L Tuning Mixture, Agilent) in IM-MS Browser (B.10.00, Agilent) with methods previously described by Stow et al. for single-field CCS calibration.^21^ Multiplexed files were demultiplexed (DeMP) using PNNL PreProcessor 4.0 (2022.02.18), with the IM sampling density increased using the five-to-one data point interpolation setting in PreProcessor (DI5, Table S4).^19,43^ The heavy-labeled internal lipid standards were used for normalization and retention time alignment. Feature lists were generated using the ion mobility feature extractor (IMFE) algorithm in Mass Profiler (10.0.2, Agilent). Demultiplexed and data point interpolated files were further processed to high resolution using the High Resolution Demultiplexing Tool 2.0 (2.0.118, Agilent) with the following settings: HR processing level set to “high”, a *m*/*z* width multiplier of “6”, and the instrument function (IF) multiplier surveyed between 0.875 and 1.100 (Table S5). Lipid Annotator (1.0, Agilent) and Mass Profiler were used to analyze the features for LC-MS/MS and LC-IM-MS, respectively. Alignment tolerances for retention time, *m*/*z*, and drift time were set to ±0% + 0.3 min, 15.0 ppm + 2.0 mDa, and ±1.5%, respectively (Table S6). Feature alignment values between the three IM acquisition modes were extracted using Venny 2.1 and were visualized using PNNL’s Venn-Diagram-Plotter from the GitHub repository.^44^ Features were identified using MassHunter ID Browser (10.0) with databases generated from the LIPID MAPS Structure Database and the Unified CCS Compendium in PCDL Manager (B.08.00, Agilent).^45−48^ MetaboAnalyst 6.0 was used for significance and differential analysis of lipids in biological samples.^49^

## Results and Discussion

**Figure 1:**
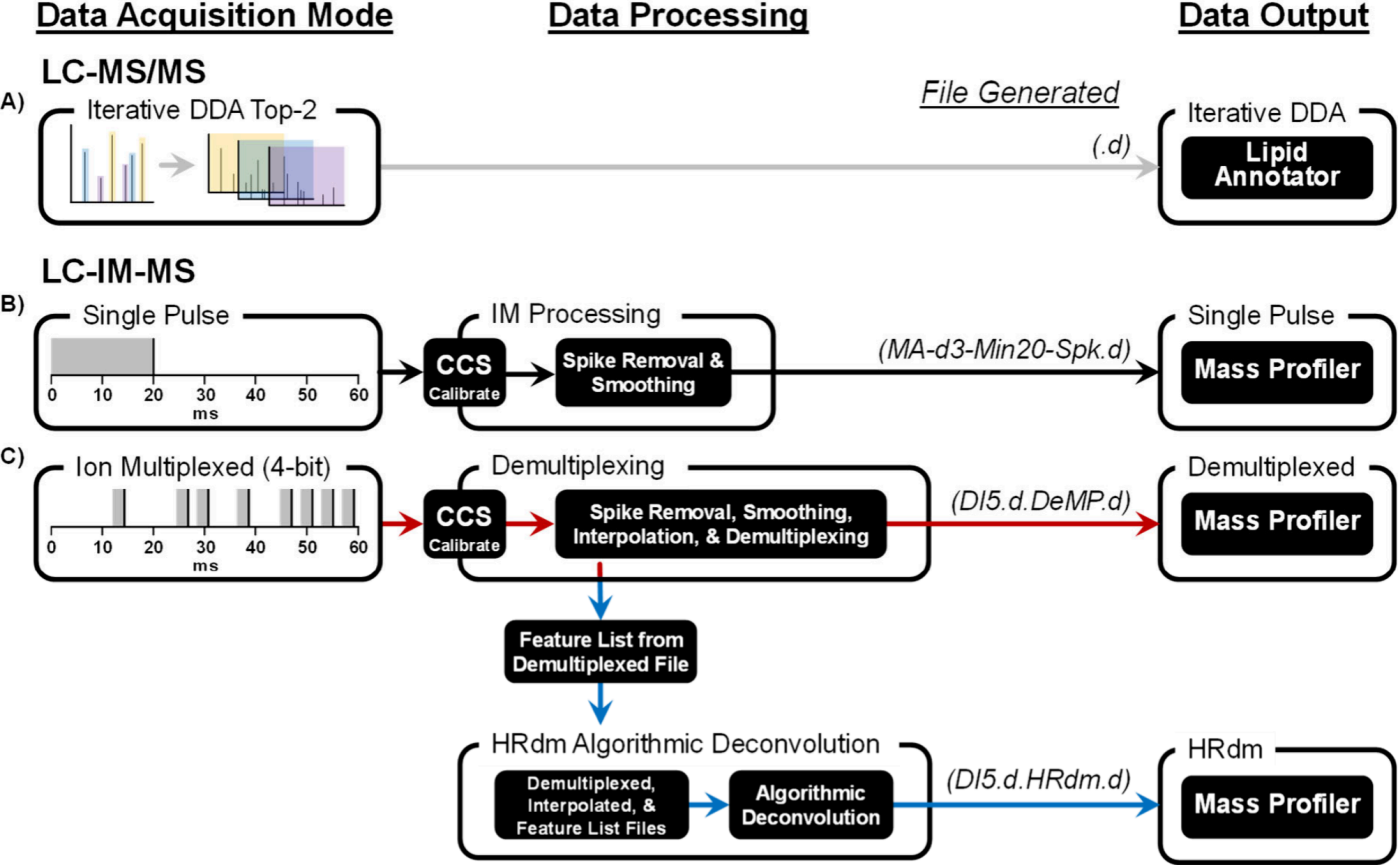
Untargeted lipidomics data processing workflows for (A) LC-MS/MS, (B) single pulse LC-IM-MS, and (C) 4-bit ion multiplexed LC-IM-MS data acquisition modes. File extensions generated during post-processing for each acquisition mode are italicized. Single pulse LC-IM-MS data was acquired for comparative analysis but is not necessary for the multiplexed workflow.

**Figure 2:**
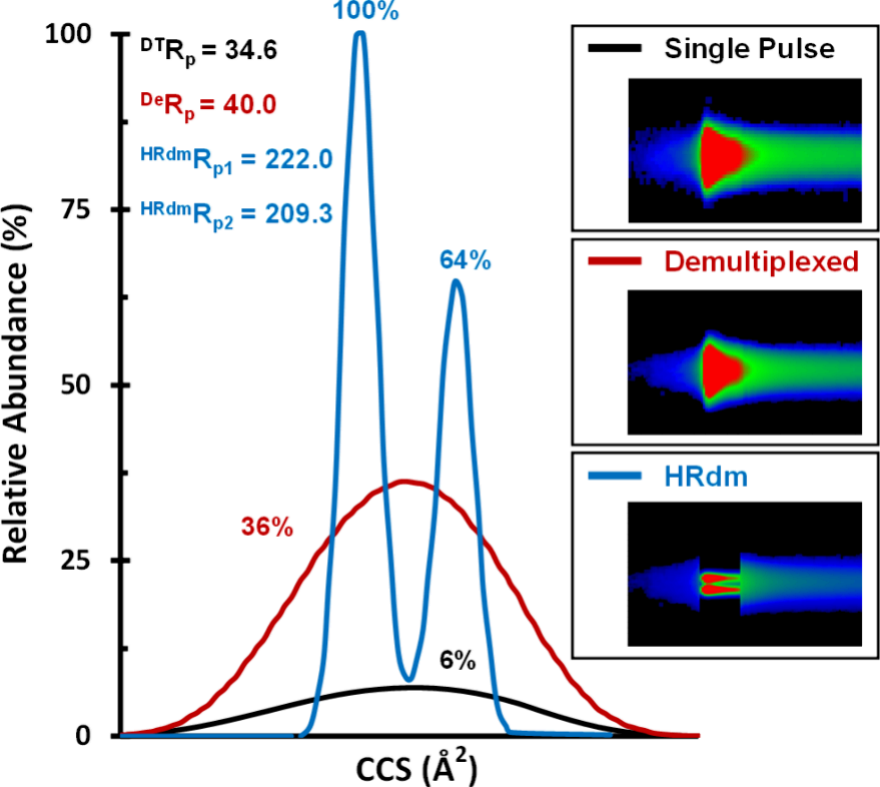
Representative data obtained for an example lipid, PC 38:6, demonstrating increased signal for demultiplexed acquisition (red trace) compared to single pulse (black trace) and increased CCS resolution following HRdm algorithmic deconvolution (blue trace). Percent relative abundances are denoted above each peak and associated resolving powers (*R*_p_) are color coded. Peaks are numbered from left to right. Corresponding heat map data (*x* axis = *m*/*z*; *y* axis = CCS) are shown in the insets.

Details of the workflows evaluated in this study are listed in Figure 1. For initial evaluation, three separate datasets were acquired in both polarities on a pooled mouse plasma lipid extract spiked with heavy-labeled exogenous lipids: a liquid chromatography-tandem mass spectrometry (LC-MS/MS) dataset, a LC-single pulse ion mobility (IM) dataset, and a LC-4-bit multiplexed IM dataset. The iterative data-dependent LC-MS/MS acquisition (Figure 1A) was used to support the lipid identifications obtained from retention time ordering and exact mass database searching. This complementary LC-MS/MS dataset was acquired for retro-identifying features in the LC-IM-MS datasets for increased confidence where appropriate. This retro-identification with LC-MS/MS was utilized in several places in the manuscript for confident identification of lipid features in the LC-IM-MS datasets, including to identify SPLASH LIPIDOMIX internal standards using Lipid Annotator and extract their retention times for abundance normalization and retention time correction for subsequent analyses in Mass Profiler, to identify the feature in Figure 2 for the single pulse, demultiplexed, and HRdm acquisitions, and to identify the SPLASH LIPIDOMIX internal standards for IF optimization in Figure S2. The conventional single pulse IM data acquisition (Figure 1B), though not necessary for the multiplexed workflow, was acquired solely for comparative analysis. Multiplexed IM was acquired in 4-bit mode and used in two evaluations: (1) the standard demultiplexed datafiles were used in a conventional LC-IM-MS workflow (Figure 1C) and (2) the multiplexed data was processed with HRdm to deconvolute the IM dimension to high resolution but otherwise retains the identical LC and MS data layers as the standard demultiplexed files. Protocols for acquiring multiplexed data and processing the raw data into HRdm output files have been previously outlined by May et al. and differ here only in the addition of the IM data point interpolation step, which provides higher data point density that improves the peak quality of the HRdm-processed IM profile data.^30^ Without data interpolation, the resulting HRdm IM profiles are narrow but undersampled and appear triangular as opposed to more desirable normal distributions. A 5-point data interpolation was used here to ensure sufficient data density was available for HRdm to utilize across the full mass and mobility range of the untargeted datasets, though we note that a focused study on the performance of different data interpolation settings was not conducted. Of note from the different workflows summarized in Figure 1 is that while the LC-MS and LC-MS/MS data process is relatively streamlined, the addition of IM data necessitates the use of multiple software platforms and data processing steps, which represents a significant time investment to manually process IM data. The HRdm workflow imposes additional software and manual processing steps, notably requiring three separate input files: a data interpolated raw file (DI5.d), a standard demultiplexed file (DI5.d.DeMP.d), and an IMFE generated list of features (.csv), which HRdm utilizes to process the output file (DI5.d.HRdm.d).

A comparison of IM profiles for PC 38:6 generated from the three IM acquisition modes confirms a significant (6×) increase in sensitivity between single pulse and 4-bit multiplexing as well as a significant (∼4×) increase in the resolving power by incorporating the HRdm algorithmic deconvolution step (Figure 2).^30^ There is also a modest increase in resolving power upon implementing standard demultiplexing compared with single pulse data, presumably due to less space charge broadening as a consequence of injecting several lower density ion packets per acquisition cycle during multiplexed operation. While nearly an order of magnitude of signal is initially lost when IM is combined with LC-MS, 4-bit multiplexed operation recovers most of the signal while providing access to HRdm.^50^ Algorithmic deconvolution with HRdm can, in turn, increase the resolution of the IM dimension, shown here by resolving two peaks where initially only a single peak was observed in both the single pulse and the standard demultiplexed datasets. This additional peak observed after high-resolution deconvolution is suggested by the broad peak measured in the conventional deconvoluted profile (*R*_p_ < 50). This is evident when comparing the resolving power of the closest calibrant peak (*m*/*z* 922, *R*_p_ = 61) to the lipid peak shown in Figure 2 (*m*/*z* 806, *R*_p_ = 40) (Figure S1). While we have not validated the dataset presented in this work with another HRIM platform, similar data from lipid standards have demonstrated that lipids tend to exhibit multiple features under HRIM analysis.^14^ However, additional analytical measurements (e.g., energy-resolved MS) would need to be conducted in conjunction with HRIM to determine if these multiple peaks are lipid isomers or conformers.

The HRdm 2.0 software incorporates an instrument function (IF) multiplier option to offset instrument-specific weighting factors in the HRdm algorithmic deconvolution. The IF is directly related to the peak widths expected under normal IM analysis and thus should scale with experimental changes that affect IM resolving power (e.g., different instruments or modes of operation). Operating the instrument under different ion polarities utilizes distinct tuning parameters along the ion beam path for for positive and negative ion modes, and thus is expected to influence IM peak widths and resolution. To assess the IF multiplier in both polarities, an optimization was performed on a single biological sample of lipid extract from mouse plasma (Figure S2). Identifications for IF optimization were manually confirmed by MS/MS in Lipid Annotator for high-confidence annotation. For this analysis, IF multiplier values of 0.950 and 0.875 for positive and negative modes, respectively, were found to provide optimal separation for several examples, including a biologically relevant lipid (PC 34:1) and several classes of heavy-labeled lipid standards. In the positive mode results, the peak resolution differences between IF values of 0.900 and 0.950 were found to be minor across several example classes of lipids, while peak broadening increased at values of less than 0.900. In contrast, the negative mode required an IF value of 0.875 for peak separation in several cases. Evaluation of the HRdm IF parameter has not yet been reported in the literature, and here, we find different optimal values across the two ion polarity operations, which are lower than the default IF setting of 1. As such, these optimal IF multiplier values were used for subsequent untargeted HRdm analyses for each respective ionization polarity mode (positive mode IF = 0.950; negative mode IF = 0.875). To provide context to this difference in IF values found across the different instrument operational polarities, the resolving power of the *m*/*z* 922 tune mix ion in positive mode and the comparable *m*/*z* 1033 in negative mode were investigated. For these tune mix ions, the resolving powers are 60.5 and 51.1 for positive and negative ionization modes, respectively, indicating that the instrument’s measured ion mobility peak widths differ between polarities, and therefore, it is reasonable that a different instrument function is needed for HRdm deconvolution between polarities. However, this observation has not been previously reported, and thus, additional studies performed on other drift tube instruments could lend more information on the reason for this processing phenomenon. Such a cross-platform study could also identify IF values that are correlated to the observed resolving powers of tune mix ions, negating the need to perform an HRdm optimization study for each instrument and mode utilized.

**Figure 3:**
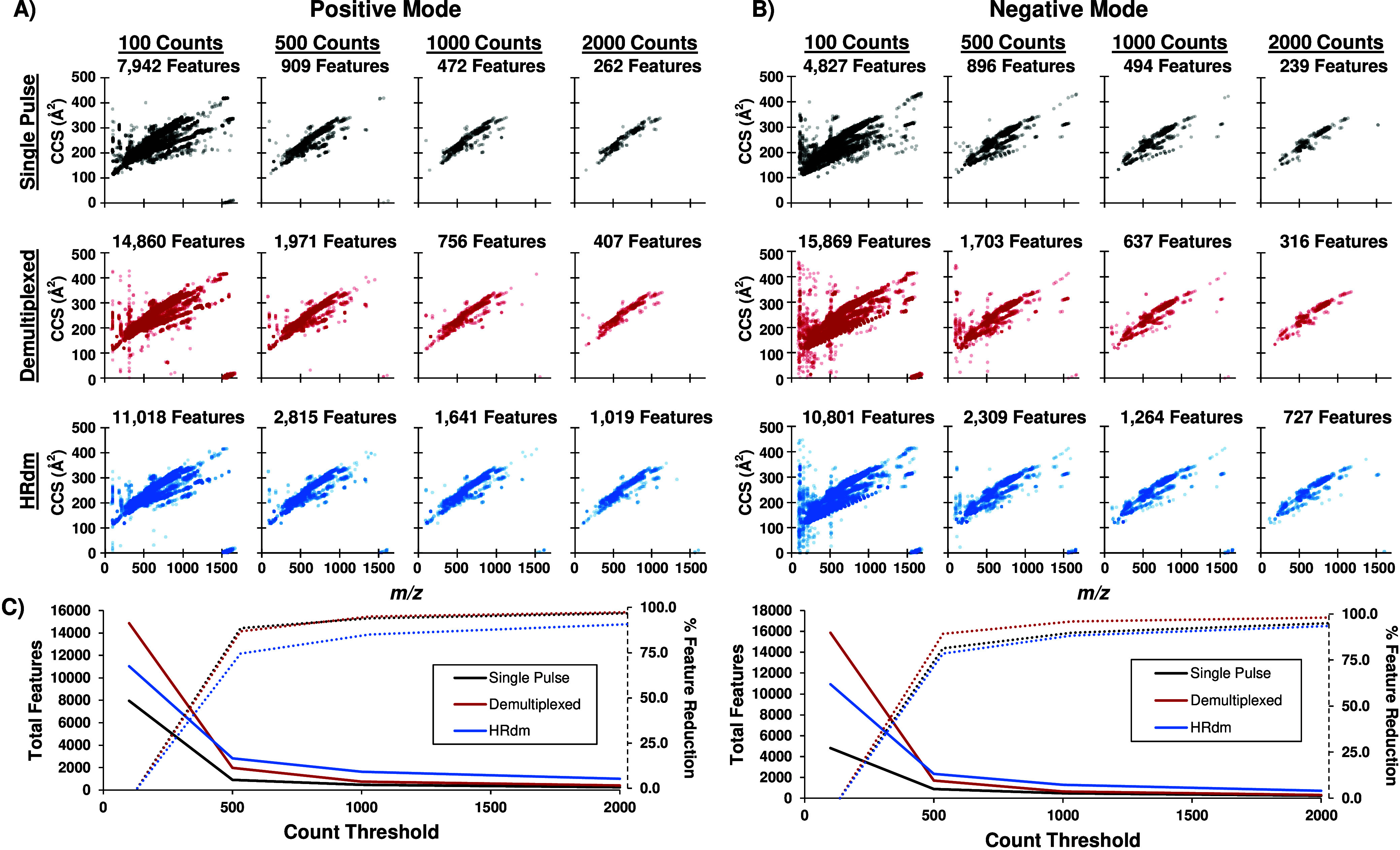
Conformational space plots (CCS vs *m*/*z*) illustrating the total features found using the IMFE algorithm for single pulse, demultiplexed, and HRdm at thresholding intensities of 100, 500, 1000, and 2000 counts in (A) positive and (B) negative ionization modes. (C) Total features (solid line) and percent features reduced (dashed line) for both polarities across the various ion count thresholds evaluated for single pulse (black), demultiplexed (red), and HRdm (blue) data files.

Generally, there are several processing steps to consider for analyzing untargeted lipidomics datasets, including filtering, feature detection, and feature alignment.^51^ An initial filtering step aims to remove baseline noise so that feature detection can identify true ion signals originating from molecules based upon the presence of an isotopic envelope and ultimately reduce false-positive detection. In this context, feature detection identifies molecular features and does not include a count of the isotopes for each ion signature (deisotoped)—for LC-IM-MS, the resulting molecular features represent triplets of the retention time (RT), *m*/*z*, and CCS.^50^ The feature alignment step seeks to align features for the same molecules across different samples and data files on the basis of measurement tolerances set for each of the three data dimensions (RT, *m*/*z*, and CCS). As such, a filtering threshold for ion signal is useful for optimizing the analysis of untargeted HRdm datasets. To determine the optimal thresholding counts for untargeted HRdm data, an evaluation of 100, 500, 1000, and 2000 count thresholds was gauged for the number of total features in Mass Profiler compared across the single pulse, demultiplexed, and HRdm data files. First, total features were assessed for each LC-IM-MS mode (single pulse, demultiplexed, and HRdm) to provide an overview of the global changes in feature counts associated with each mode. A solvent blank was used for feature subtraction, and the following values presented here are unique to the pooled sample of mouse plasma. The results of this total feature evaluation for different count thresholds are provided in Figure 3. General observations to note here are that at a conservative threshold of 100 counts, there are significantly more features associated with the multiplexed datasets (demultiplexed and HRdm) than the single pulse data, which indicates both the presence of real signal due to increased sensitivity and contributions from multiplex-specific data deconvolution artifacts that are introduced during the inverse transform of the data (notable in the low *m*/*z* range as uncorrelated “streaking” across the full CCS range). The feature plot summaries (Figure 3C) reflect this observation of more features present in both multiplexed datasets at 100 counts; however, standard demultiplexed (red trace) exhibits a higher feature number than the HRdm dataset (blue trace), which contradicts the expectation that more resolved features should be present in the higher resolution HRdm data. Implementing a higher count threshold of 500, however, significantly decreases the overall feature numbers (by 83.7% on average) for all modes and polarities, and the expected magnitude of features (HRdm > demultiplexed > single pulse) is observed, which suggests that a large number of the features detected at 100 counts originate from a lower quality signal and data artifacts, that is, the increased signal from IM multiplexing is expected to increase the total features; however, at 100 counts, these features are primarily raising the baseline of chemical and spectral noise, and the majority are thus irrelevant to lipid analysis. At higher count thresholds of 1000 and 2000, the expected relative ordering of features remains consistent with fewer features being rejected for each progressive increase in the threshold. Increasing the threshold from 500 to 1000 counts reduces the number of features by 50.2% on average, while an increase from 1000 to 2000 counts corresponds to an average decrease of 43.9% of the total number of features (Figure 3C and Table S7). Here, a 1000-count threshold was considered optimal for producing a high-quality untargeted lipidomics dataset for HRdm. Previous literature has used a 500-count threshold for single pulse untargeted IM lipidomics; however, our current results suggest that a 1000-count threshold provides a higher quality dataset for HRdm lipidomic analysis and was therefore used for assessing the individual biological samples for an untargeted case study with HRdm.^16^

**Figure 4:**
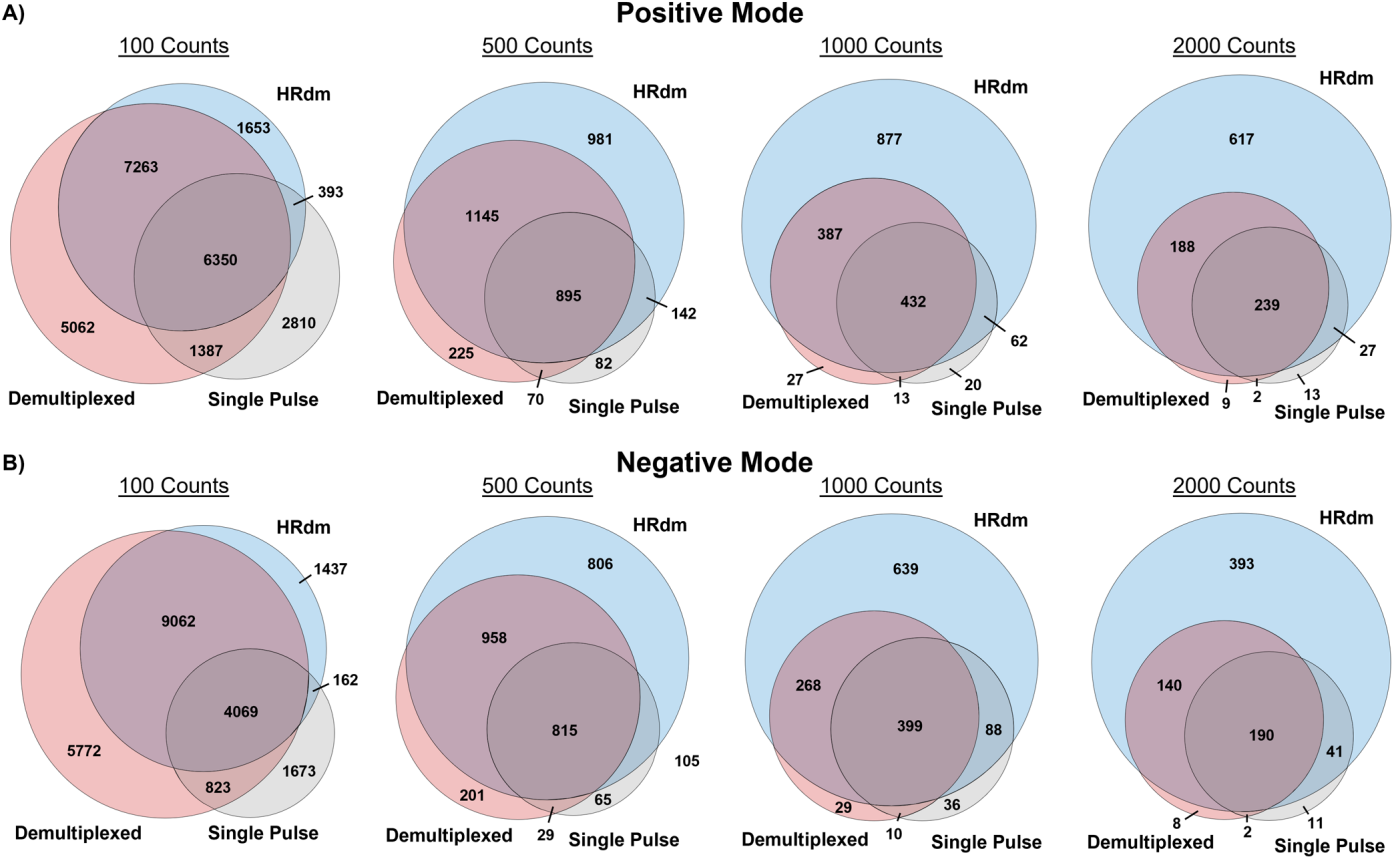
Venn diagrams demonstrating feature overlap among the three ion mobility acquisition modes (single pulse, demultiplexed, and HRdm) for different threshold intensities. Feature alignment results for both the (A) positive and (B) negative modes are shown.

Feature alignment was next conducted across the three acquisition modes to assess the overlap in the features observed. Feature similarity across all three IM acquisitions was hypothesized to converge at higher threshold counts due to the removal of signal from chemical and spectral noise present at lower threshold counts. Additionally, since HRdm differs from the demultiplexed dataset only in the resolution of the IM dimension, it was expected that most of the demultiplexed features would also be observed in the HRdm dataset. Furthermore, HRdm should exhibit the highest number of unique features since this IM acquisition mode operates with the highest sensitivity and resolution. The related features observed across the three modes are summarized in Figure 4 for both ion polarity datasets. At the lower signal filtering thresholds (100 and 500 counts), there are a significant number of features unique to each acquisition mode and relatively few features are unique to HRdm. However, as the signal threshold is increased (left to right), the Venn diagrams exhibit the expected outcomes, namely, a large amount of features detected in single pulse mode are also observed in the two multiplexed datasets, and HRdm exhibits the largest number of total features overall, many of which are unique features. However, potentially spurious features can be introduced through feature finding across multiple acquisition types and thresholding cutoffs. For example, a cursory look at these 13 and 11 unique features for positive and negative mode, respectively, at the 2000 count threshold in single pulse acquisition mode (Figure 4) were mostly due to degenerate feature selection and concatenation between acquisition modes and count thresholding cutoffs, leading to fractional differences in the measurements that resulted in the same feature being identified as distinct across the datasets. Overall, features unique to HRdm are consistently higher in number than is observed in both demultiplexed and single pulse datasets at thresholds of ≥500 counts in both polarities (i.e., 981 unique features in HRdm positive mode, 225 unique features in demultiplexed mode, and 82 unique features in single pulse). Furthermore, there was a greater amount of overlap across all three IM acquisitions, and convergence of feature alignment was observed at ≥1000 counts. This indicates that HRdm captures a high percentage of similarity to both of the other IM acquisitions while resolving additional features for lipidomic analysis.

Using the ion multiplexed LC-HRIM-MS workflow developed here, an untargeted lipidomics case study of biological samples was investigated to assess the impact of lipid isomer separation capabilities for providing biologically relevant information. Murine models with functional deficiency of a cell membrane flippase, ATP10D, which is suggested to be involved in dyslipidemia, were explored.^40,52,53^ Lipids were extracted from the plasma of three mice expressing functional alleles of *Atp10D* (+/+) and three mice expressing dysfunctional alleles (−/−). Positive and negative mode LC-HRIM-MS data were acquired, and significant and differentially expressed features were identified with high confidence by exact mass and CCS using the Conformational Lipid Atlas hosted in the Unified CCS Compendium (UCC).^12,47^ Features that were not identified by the UCC were iteratively processed through the LIPID MAPS Structure Database (LMSD) for exact mass identifications.^45,46,48^ The demultiplexed dataset in positive mode had a total of 844 features across the 6 mouse samples with 525 features (62%) identified based upon LMSD and 115 high confidence hits (14%) from the UCC. Demultiplexed negative mode exhibited fewer total features than positive mode with 454 total negative mode features but similar percentages of database matches as observed in positive mode, with 259 LMSD hits (57%) and 66 UCC identifications (15%). After HRdm deconvolution, the number of positive mode features increased by 2.5-fold, with a total of 2178 features with 1370 identifications from LMSD (63%) and 228 identifications from the UCC (10%). HRdm processing of the demultiplexed datasets in negative mode also resulted in a 2.5-fold increase, to 1135 total features with 652 LMSD hits (57%) and 127 UCC identifications (11%). Of note from these results is that HRdm processing yielded a similar (2.5-fold) increase in features in both ion modes with similar percentages of exact mass hits (57–63%), though fewer features were identified with CCS searching via the UCC (∼10% HRdm vs ∼14% demultiplexed), likely a result of the mismatch in IM resolution between the UCC (*R*_p_ ≈ 50) and the HRIM values generated in this current workflow (*R*_p_ ≈ 200). Also of note, the databases differ in size by a factor of 48 with the LMSD containing >48 000 lipid entries and >1000 lipid or lipid-like structures for the UCC, which likely contributes to the higher percentage of hits.

**Figure 5:**
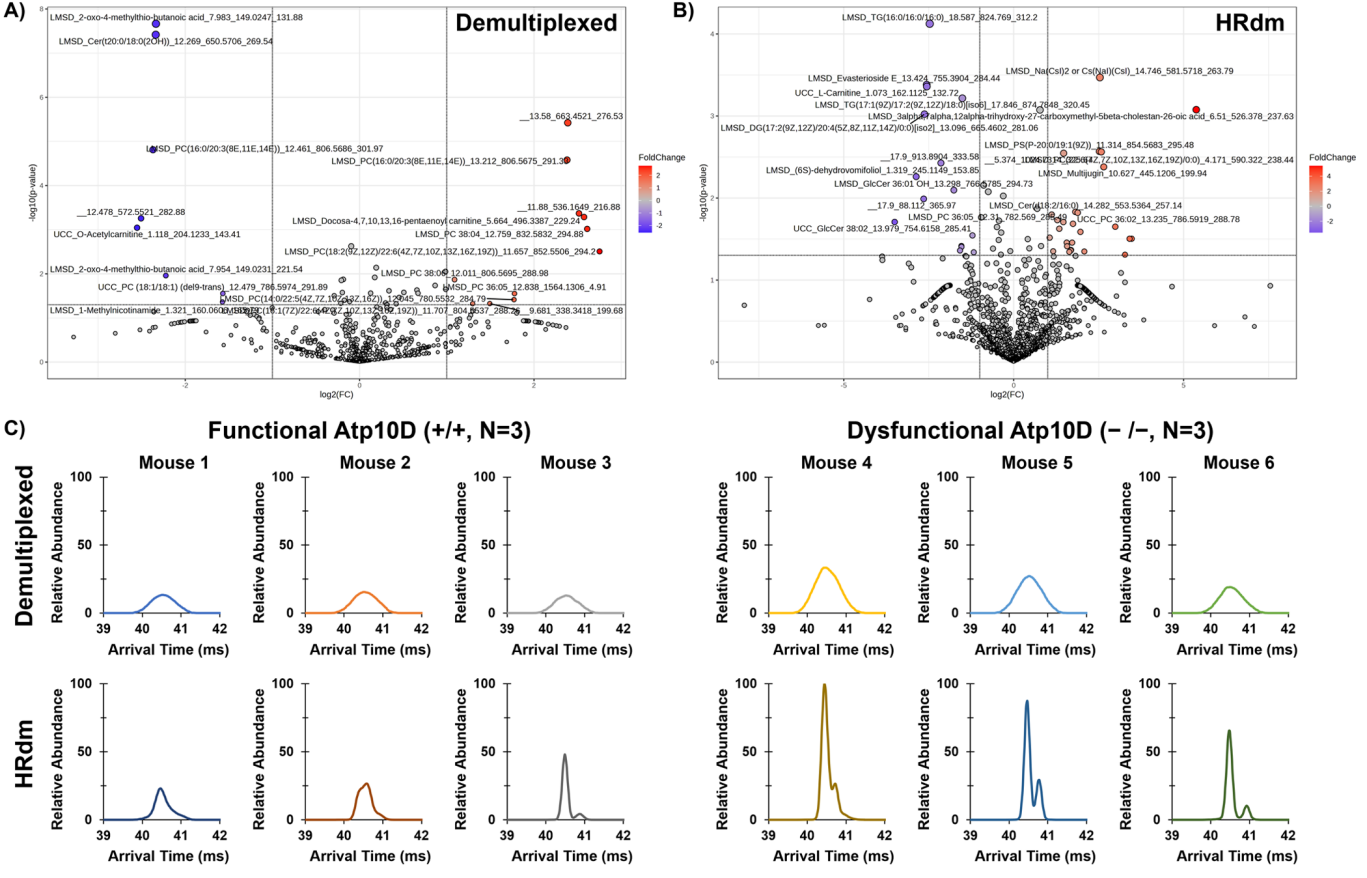
Volcano plots from MetaboAnalyst 6.0 between functional and dysfunctional ATP10D mouse models for (A) standard demultiplexed and (B) HRdm acquisition modes in positive mode with annotations including the identification database, lipid identification, retention time, mass-to-charge, and CCS value denoted above the features with a *p* value < 0.05 and a log_2_-fold change > 2. (C) Arrival time distributions for an example lipid, LMSD_PC 36:5_12.31_782.569_285.39, for three ATP10D functional mice (+/+) and three ATP10D dysfunctional mice (−/−) for both demultiplexed and HRdm with relative abundance normalized to the maximum ion signal across all six mice and both acquisition modes.

Significance (*p* value < 0.05) and fold change (log_2_ fold change > 2) between functional and dysfunctional *Atp10D* mouse models were next evaluated using MetaboAnalyst 6.0.^49^ Features that were not detected below a 1000-count threshold were imputed using a missing data function in MetaboAnalyst 6.0, removing features with >70% missing values across the samples and estimating the remaining missing values by replacing them with 1/5 of the minimum positive value of each variable. No data filtering or normalization was used in MetaboAnalyst 6.0 as the data was already prefiltered and aligned in Mass Profiler. The resulting volcano plots were analyzed for significant features in demultiplexed and HRdm across both polarities (Figure 5A and Figure S3). In the positive mode demultiplexed data, 11 features were found to be significantly up in the dysfunctional *Atp10D*^*–/–*^ mouse model and 8 features were significantly down (Figure 5A), while in negative mode, 4 features were significantly up and 11 features were significantly down (Figure S3). The positive mode HRdm dataset exhibited 27 significantly up features and 15 features significantly down (Figure 5B), while HRdm negative mode had 7 significantly up features and 23 significantly down features (Figure S3). An increase in total significant features was observed for HRdm in both polarities compared to that of demultiplexed as a result of the increase in total features from HRdm analysis. In the HRdm dataset, an example lipid identified as PC 36:5 was found to be significantly up in the *Atp10D*^*–/–*^ mice (Figure 5C), with two peaks being resolved in all three samples, while only a single peak was observed from two *ATP10D*^*+/+*^ mice. These doublets are not observed in the demultiplexed data, indicating that a possible isomeric feature may be present in the plasma from the *Atp10D*^*–/–*^ mice. The plasma from one *Atp10D*^*+/+*^ mouse exhibited a small doublet peak for PC 36:5, however, this second peak was below the 1000-count threshold and thus was not included in the differential analysis. These doublet peaks for PC 36:5 in HRdm correlate to CCS values of 285.39 and 287.78 Å^2^, corresponding to a minor 0.83% difference in CCS (Table S7). This example feature illustrates the utility of incorporating the HRdm deconvolution strategy into untargeted lipidomics and has the potential to reveal additional features significant to biological studies at the isomeric level. While the workflow outlined here can be used to identify significant features using HRIM, we note that the computational tools needed to automate this process is currently lacking, and manual intervention is thus required for discovering additional significant isomeric features and for interpretation of the results for biological context.

## Conclusions

The HRdm integrated LC-IM-MS workflow described here demonstrates promise for deployment into untargeted lipidomic analyses to provide increased feature counts and improved separation. Additionally, HRdm retains the high-precision CCS measurements that are inherent to the drift tube technique while providing additional features that are only observed with HRIM analysis. The critical analysis of HRdm parameters and signal thresholds described in this report provides a practical basis for integrating these advanced multiplexed ion mobility modes into established untargeted workflows. Once established, these operational conditions were successfully applied to lipidomic extracts of a murine lipid dysregulation model and identified a significant lipid as exhibiting two resolved peak features unique to HRIM which are likely a result of multiple isomeric lipid species present at the same *m*/*z* and retention time.

The biological implications of isomer-specific untargeted omics are potentially far reaching; however, the analytical capabilities to generate and interrogate the datasets are still emerging. Current limitations in the computational tools available and, importantly, the ability to streamline the workflows incorporating multiple software packages represent a barrier for broader adoption of advanced analytical capabilities such as multidimensional LC-IM-MS and LC-IM-MS/MS. Whereas there are now several HRIM offerings, including HRdm, now being made broadly available to researchers (e.g., SLIM, cIMS, TIMS), the results of this current investigation with HRdm hint at great promise for deploying these capabilities into untargeted discovery but also underscore a current need to support these workflows with databases of HRIM-generated CCS measurements. Moreover, MS/MS can be coupled with HRdm, both in a conventional DIA experiment as recently demonstrated by Xu et al.^54^ and in a data-independent, all-ion post-mobility fragmentation strategy which can yield comprehensive MS/MS coverage for improved molecular annotation. As these HRIM databases and software solutions are developed in future work, widespread adoption of HRIM technologies is expected to make a defining impact in the omic sciences.

## Supporting Information Available

Data acquisition, data processing, and data analysis parameters; additional figures for HRdm instrument function multiplier optimization and differential lipid analysis in negative mode (PDF)

## Abbreviations

CCS: collision cross section
cIMS: cyclic ion mobility spectrometry
DT: drift time
DTIMS: drift tube ion mobility spectrometry
HRdm: high-resolution demultiplexing
HRIM: high-resolution ion mobility
IM: ion mobility
LC-MS: liquid chromatography-mass spectrometry
LC-MS/MS: liquid chromatography-tandem mass spectrometry
LC-IM-MS: liquid chromatography-ion mobility-mass spectrometry
LC-IM-MS/MS: liquid chromatography-ion mobility-tandem mass spectrometry
LMSD: Lipid Maps Structure Database
MS: mass spectrometry
*m*/*z*: mass-to-charge ratio
PC: phosphatidylcholine
RT: retention time
SLIM: structures for lossless ion manipulation
TIMS: trapped ion mobility spectrometry
UCC: Unified CCS Compendium
