# Supplementary material for: An Untargeted Lipidomics Workflow Incorporating High-Resolution Demultiplexing (HRdm) Drift Tube Ion Mobility-Mass Spectrometry
Source: J Am Soc Mass Spectrom. 2024 Sep 14;35(10):2448–57. doi: 10.1021/jasms.4c00251 (PMC11450926; doi:10.1021/jasms.4c00251)
Supplement: Supplementary file 1 — js4c00251_si_001.pdf [file js4c00251_si_001.pdf]

**Supporting Information**

# An Untargeted Lipidomics Workflow Incorporating High-Resolution Demultiplexed (HRdm) Drift Tube Ion Mobility-Mass Spectrometry

David C. Koomen,[†] Jody C. May,[†] Alexander J. Mansueto,[‡] Todd R. Graham,[‡] and John A. McLean [†,*]

[†] Center for Innovative Technology, Department of Chemistry, Vanderbilt University, Nashville, Tennessee 37235, United States
[‡] Department of Biological Sciences, Vanderbilt University, Nashville, Tennessee, 37235, United States

*Email: john.a.mclean@vanderbilt.edu

**Supplemental Figures:**

**Table S1.** Liquid chromatography parameters for untargeted lipidomics
**Table S2.** Source conditions, acquisition parameters, and drift tube settings for single pulse
**Table S3.** Source conditions, acquisition parameters, and drift tube settings for 4-bit multiplexing
**Table S4.** Parameters for PNNL Preprocessor
**Table S5.** Parameters for HRdm 2.0 software
**Table S6.** Parameters for Mass Profiler
**Table S7.** Total features and percent reduction of threshold counts in positive and negative modes
**Figure S1.** Instrument Function (IF) optimization for example biologically-relevant lipids
**Figure S2.** Volcano plots from MetaboAnalyst for demultiplexed and HRdm datasets in negative mode
**Table S8.** PC 36:5 ion signals in murine models for demultiplexed and HRdm in positive mode

**Table S1.** Liquid chromatography parameters for untargeted lipidomics.

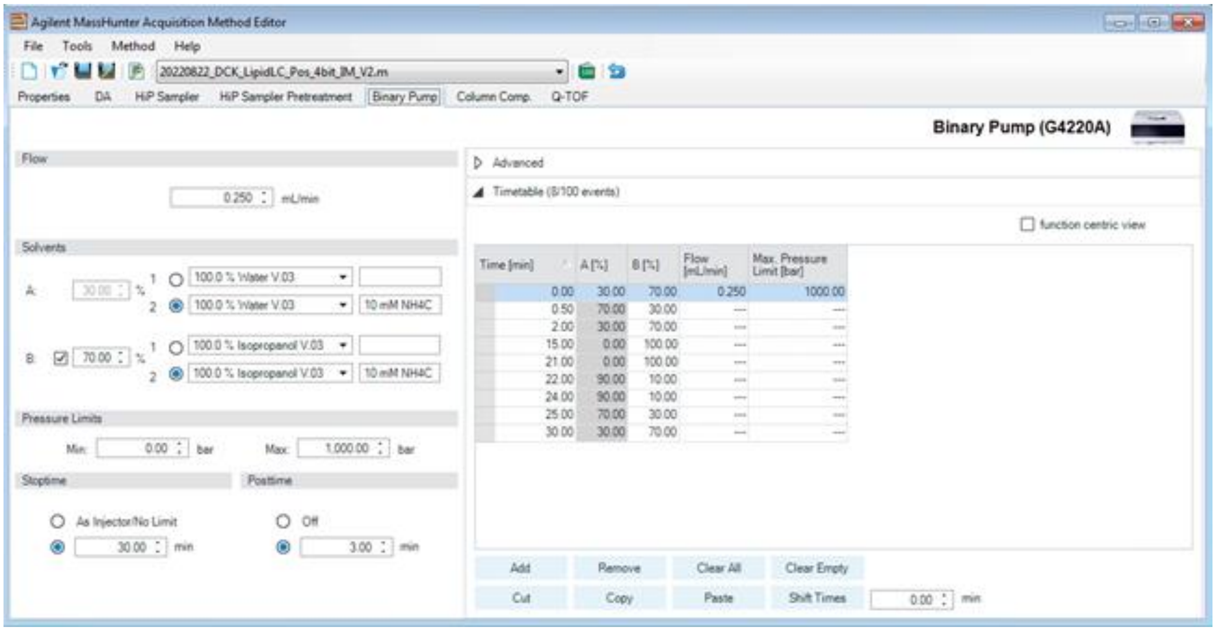

**Table S2.** Ion mobility single pulse source **(A)** and acquisition **(B)** parameters.

**(A)**

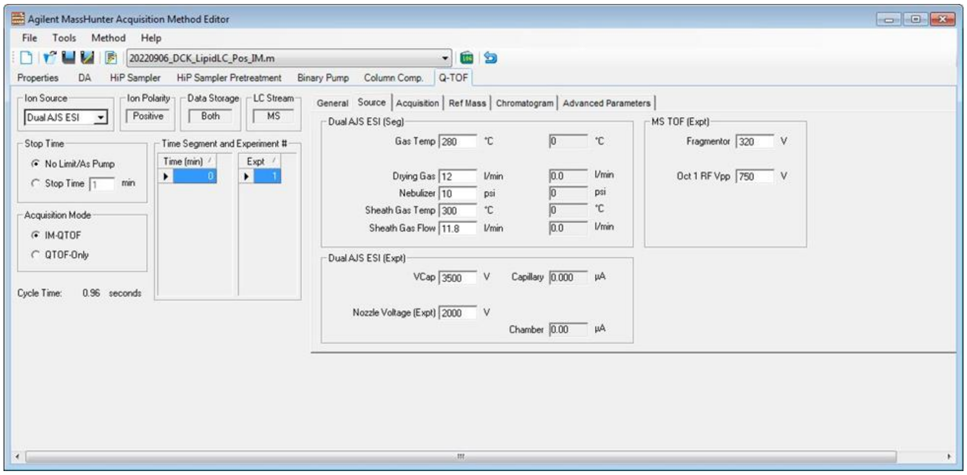


**(B)**

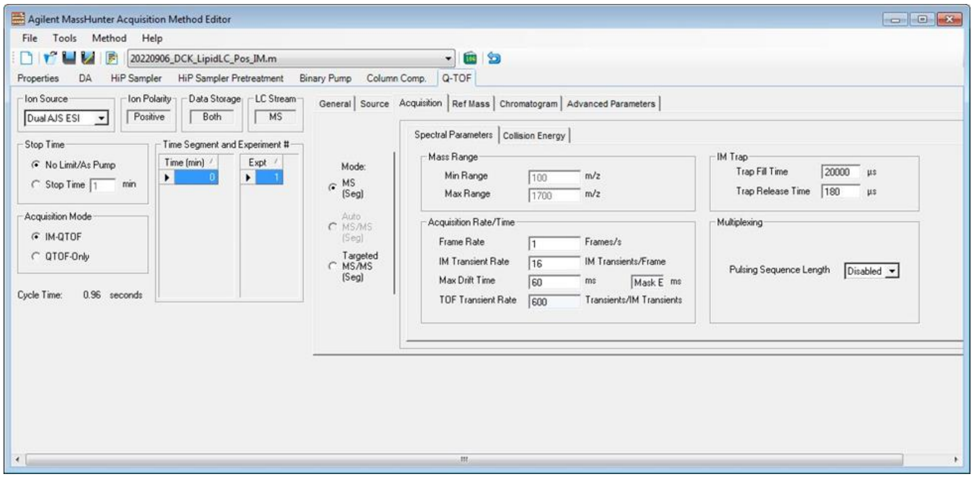

**Table S3.** Ion mobility 4-bit multiplexing source **(A)** and acquisition **(B)** parameters.

**(A)**

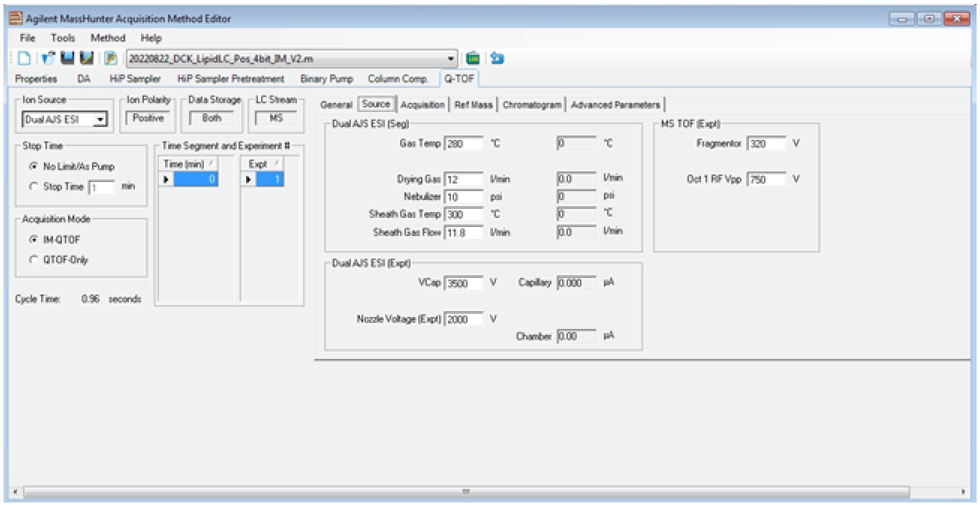


**(B)**

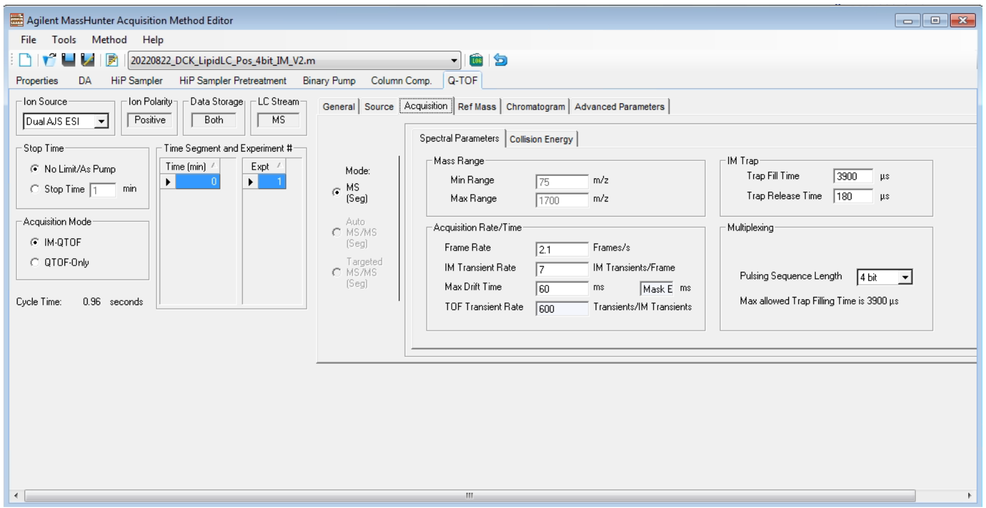

**Table S4.** PNNL PreProcessor parameters for single pulse **(A)** and 4-bit multiplexed **(B)** data.

**(A)**

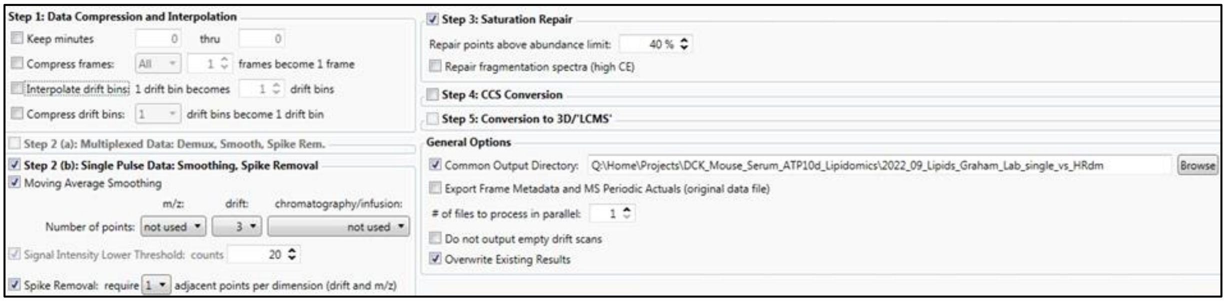


**(B)**

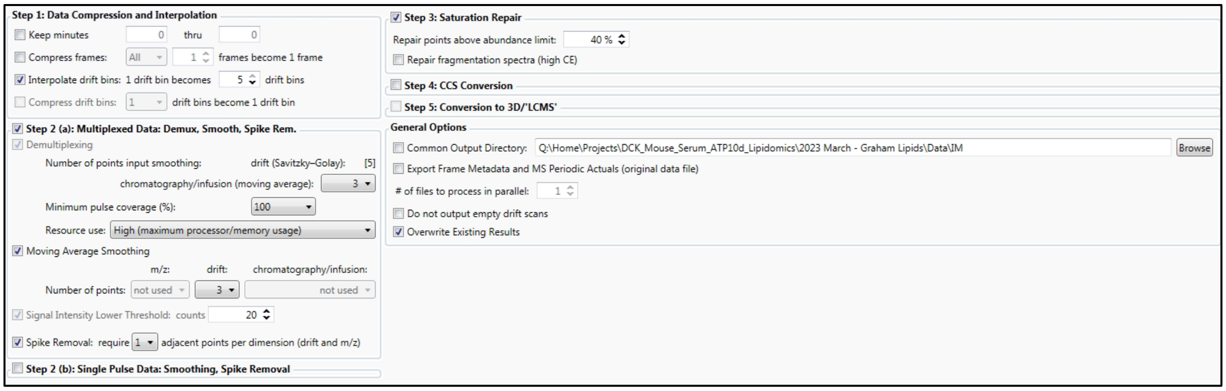

**Table S5.** HRdm 2.0 parameters.

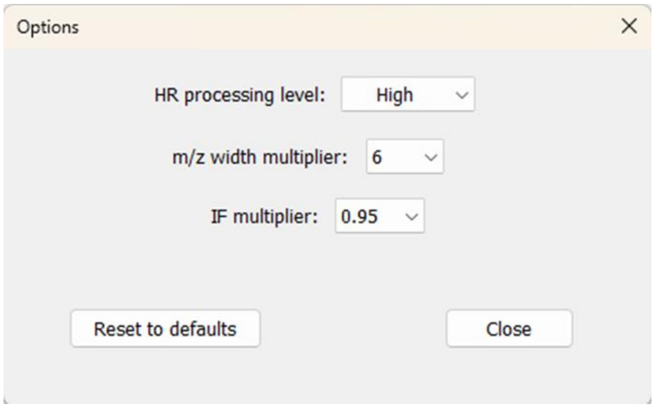

**Table S6.** Mass Profiler parameters for positive mode. Spiked-in internal standards from SPLASH LIPIDOMIX (15:0-18:1(d7) PC (*m/z* 753), 18:1(d7) LPC (*m/z* 529), 18:1(d9) SM (*m/z* 738), and 15:0-18:1(d7) PE (*m/z* 711)) were used for abundance normalization and retention time correction. Ion intensity count was changed for thresholding purposes for each file analyzed (i.e., 100, 500, 1000, and 2000 counts).

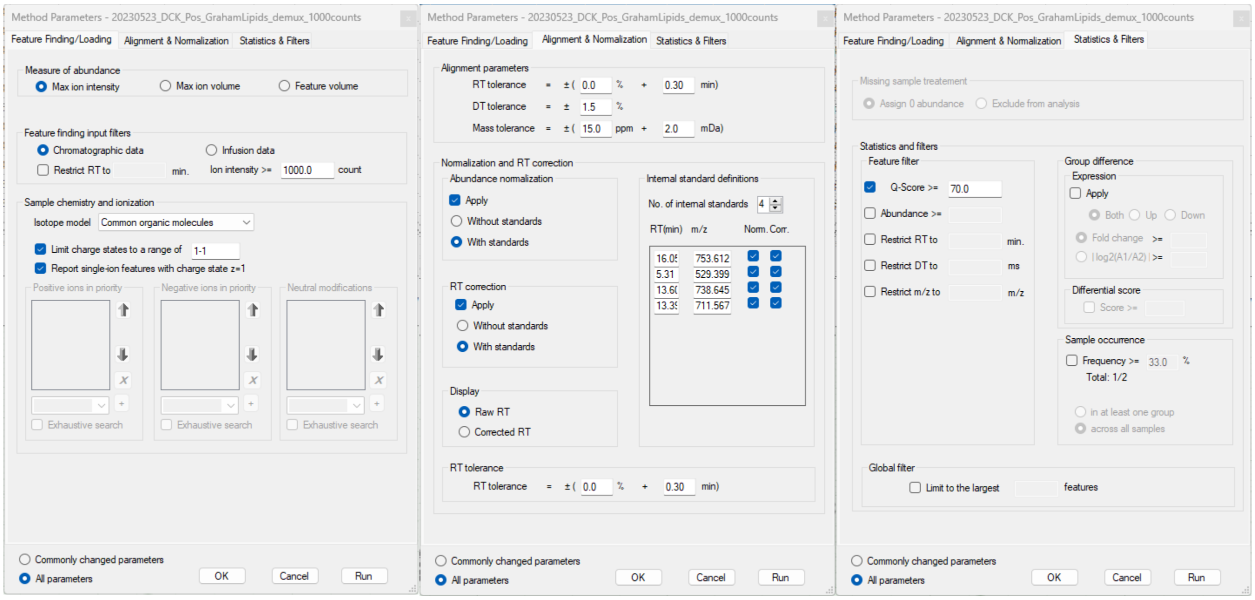

**Table S7.** Total features and percent of features reduced for threshold counts of 100, 500, 1000, and 2000 in single pulse, demultiplexed and high resolution demultiplexed (HRdm) data files.

| | | Positive Mode | | | Negative Mode | | |
|---|---|---|---|---|---|---|---|
| | Threshold Count | Single Pulse | Demultiplexed | HRdm | Single Pulse | Demultiplexed | HRdm |
| Total Features | 100 | 7942 | 14860 | 11018 | 4827 | 15869 | 10935 |
| | 500 | 909 | 1971 | 2815 | 896 | 1703 | 2343 |
| | 1000 | 472 | 756 | 1641 | 494 | 637 | 1293 |
| | 2000 | 262 | 407 | 1019 | 239 | 316 | 727 |
| % Feature Reduction | 100 | 0.0 | 0.0 | 0.0 | 0.0 | 0.0 | 0.0 |
| | 500 | 88.6 | 86.7 | 74.5 | 81.4 | 89.3 | 78.6 |
| | 1000 | 94.1 | 94.9 | 85.1 | 89.8 | 96.0 | 88.2 |
| | 2000 | 96.7 | 97.3 | 90.8 | 95.0 | 98.0 | 93.4 |

**(A)**

**Positive Mode (PC 34:1 $[M+H]^{+}$, m/z 760, RT 14.07), Sample 2037 +/+**

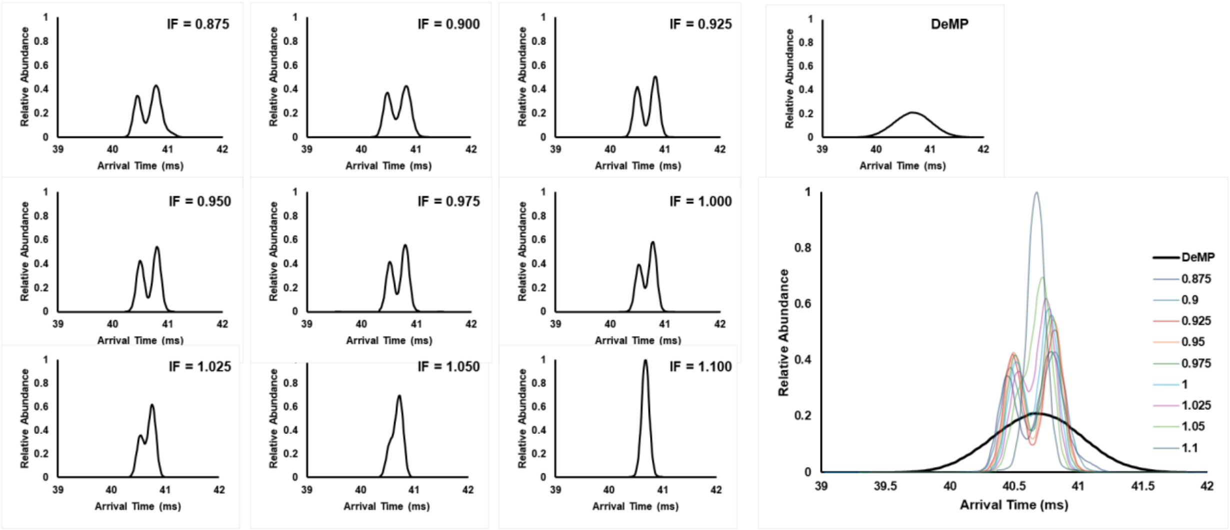


**Negative Mode (PC 34:1 $[M+CHOO]^{-}$, m/z 804, RT 14.07), Sample 2037 +/+**

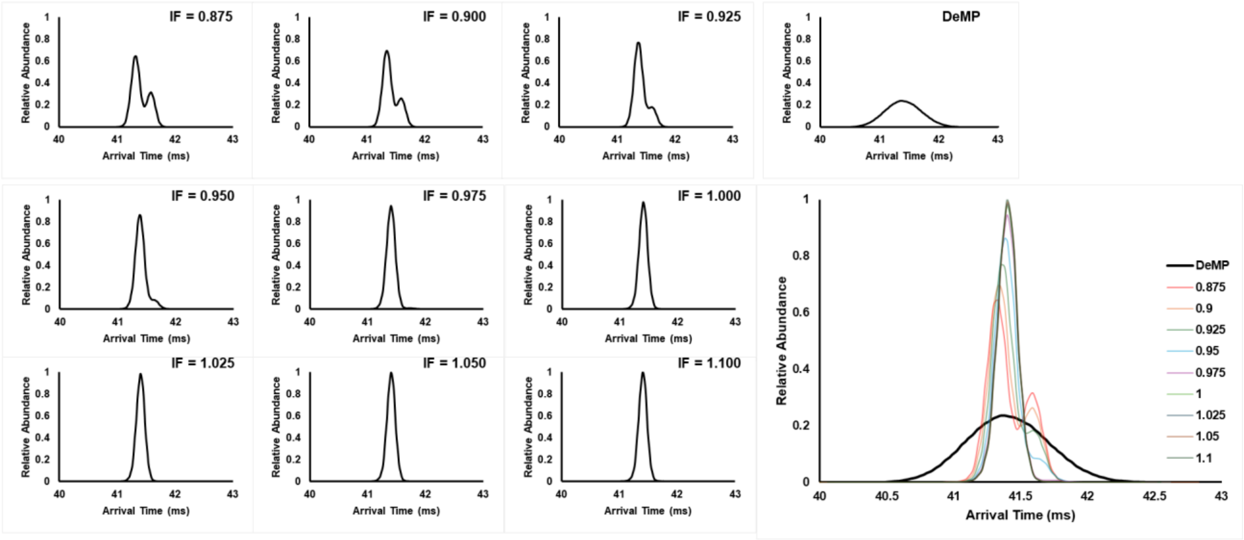

**(B)**

**Positive Mode (SM 36:2 (d9) $[M+H]^+$, m/z 738, RT 12.96), Sample 2037 +/+**

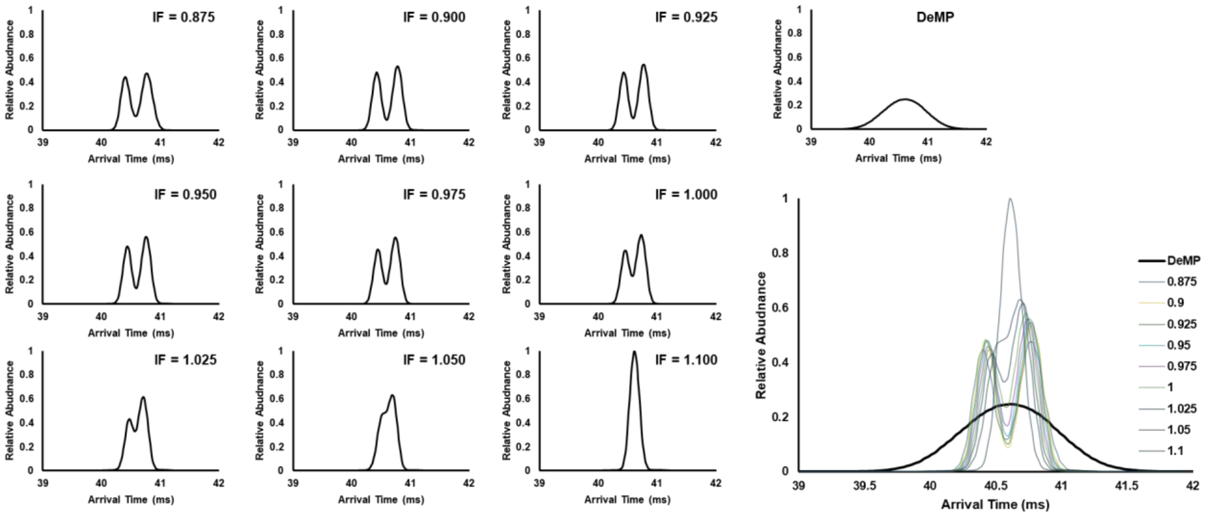


**Negative Mode (SM 36:2 (d9) $[M+CHOO]^-$, m/z 782, RT 12.96), Sample 2037 +/+**

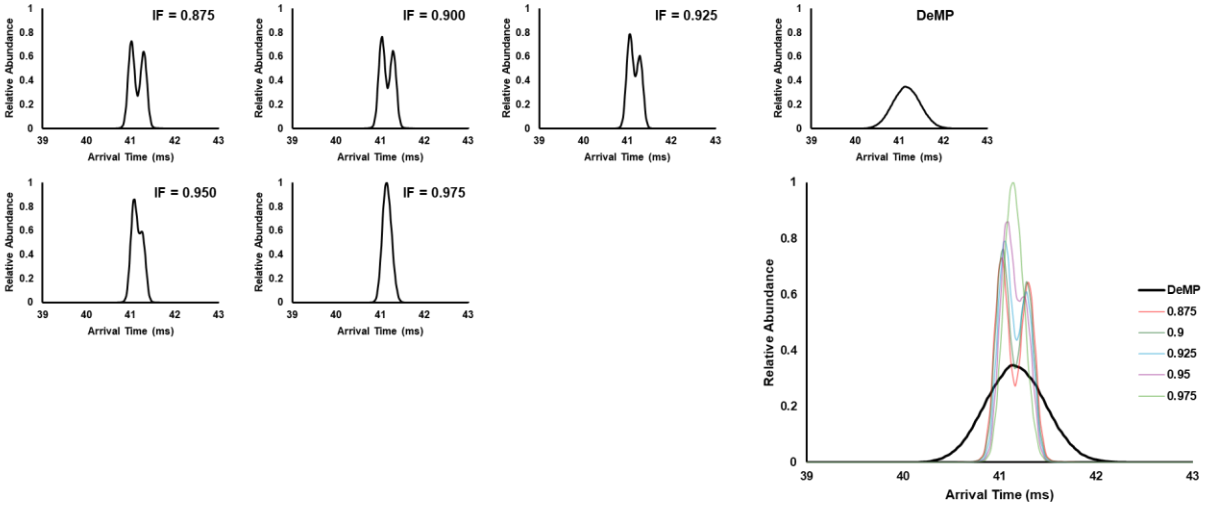

**(C)**

Positive Mode (PE 33:1 (d7) $[M+H]^+$, m/z 711, RT 13.28), Sample 2037 +/+

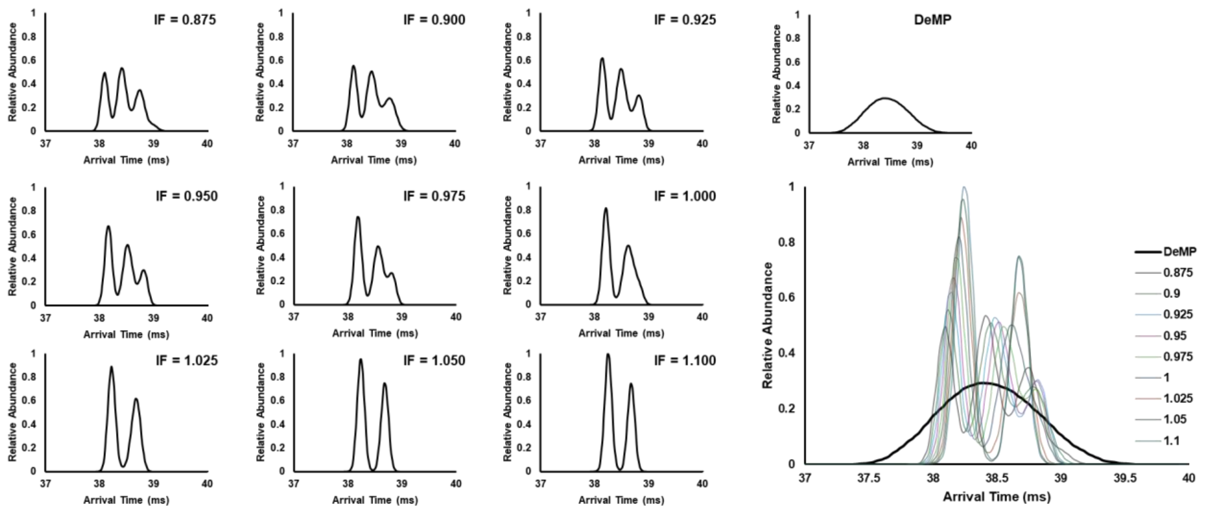


Negative Mode (PE 33:1 (d7) $[M-H]^-$, m/z 709, RT 13.28), Sample 2037 +/+

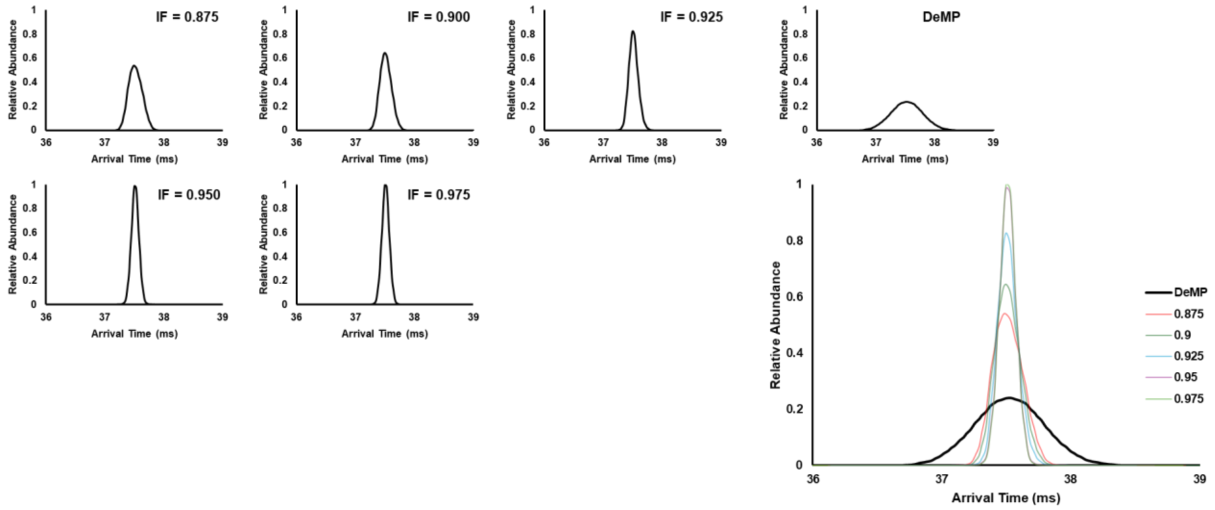

**(D)**

**Positive Mode (PG 33:1 (d7) $[M+H]^+$, m/z 759, RT 12.47), Sample 2037 +/+**

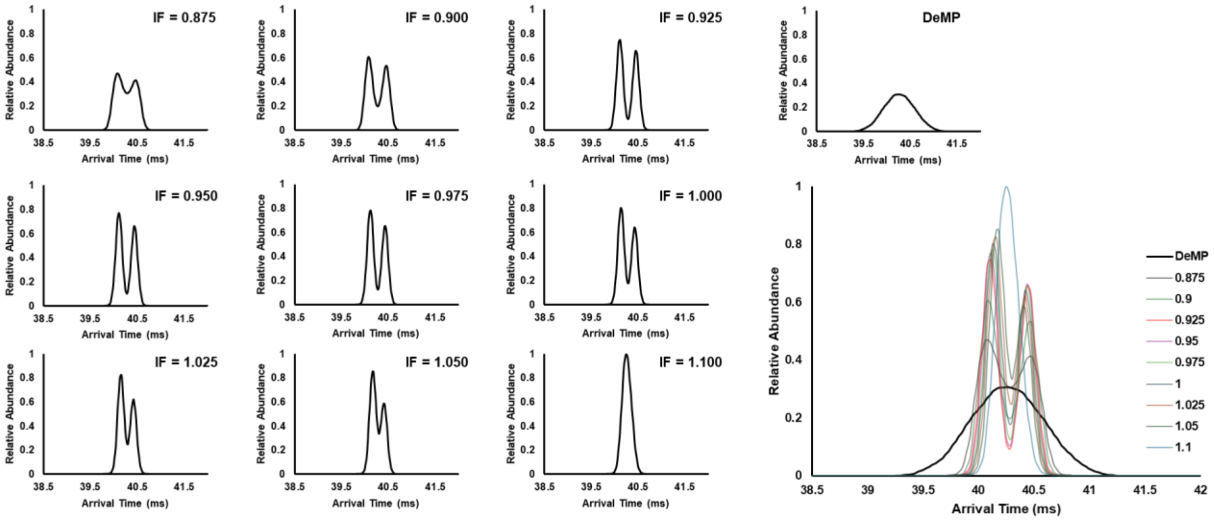


**Negative Mode (PG 33:1 (d7) $[M+CHOO]^-$, m/z 750, RT 12.47), Sample 2037 +/+**

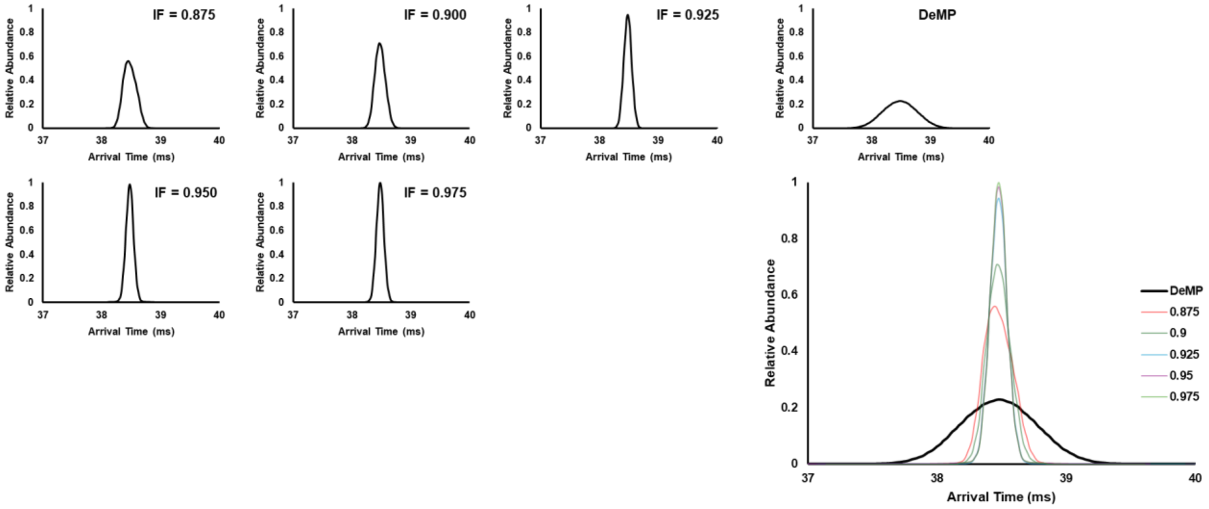

**(E)**

Positive Mode (LPC 18:1 (d7) $[M+H]^+$, m/z 529, RT 5.17), Sample 2037 +/+

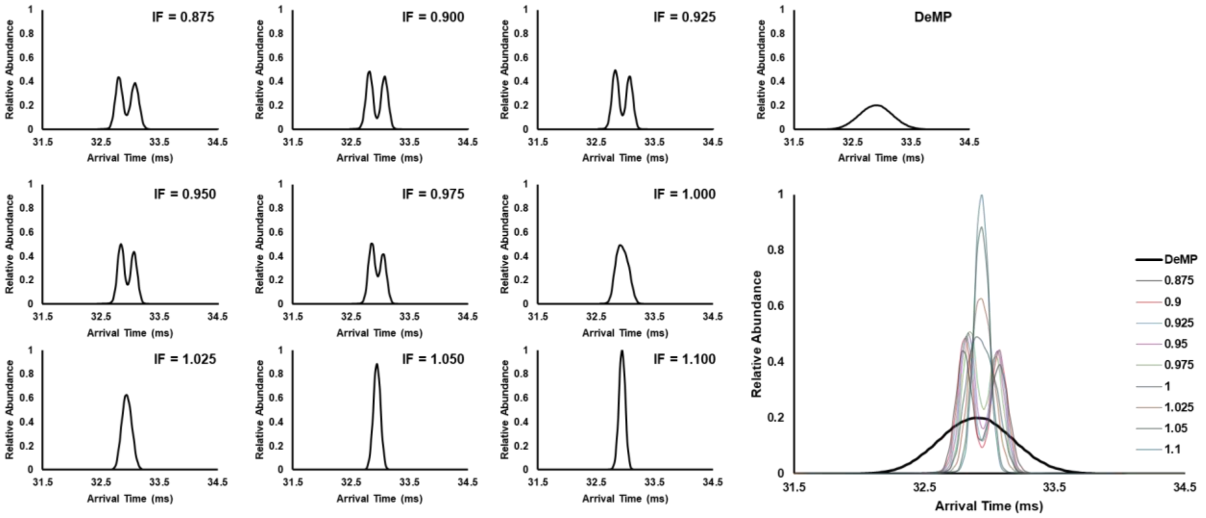


Negative Mode (LPC 18:1 (d7) $[M+CHOO]^-$, m/z 573, RT 5.41), Sample 2037 +/+

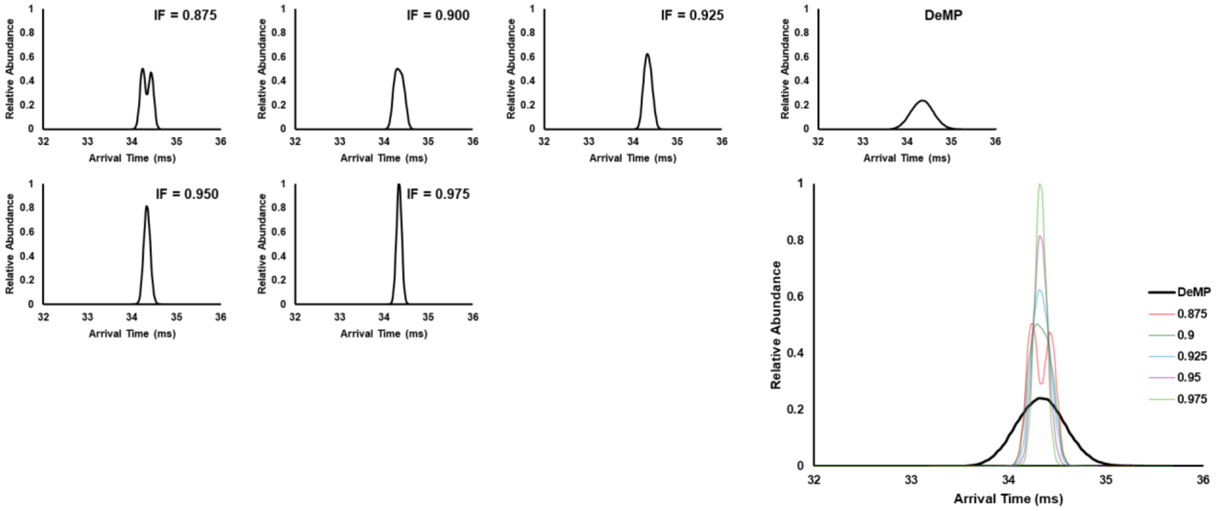


**Figure S1.** Instrument function optimization for an example mouse sample, 2037, for example lipids, **(A)** PC 34:1, **(B)** SM 36:2(d9), **(C)** PE 33:1(d7), **(D)** PG 33:1 (d7), **(E)** LPC 18:1(d7), in positive and negative ionization modes (±0.2 minute retention time). Instrument functions of 0.875, 0.900, 0.925, 0.950, 0.975, 1.000, 1.025, 1.050, 1.100 were analyzed for positive mode and 0.875, 0.900, 0.925, 0.950, 0.975 for negative mode in comparison to demultiplexed (DeMP). Relative abundance normalized to a value of 1 from the maximum value across all instrument functions.

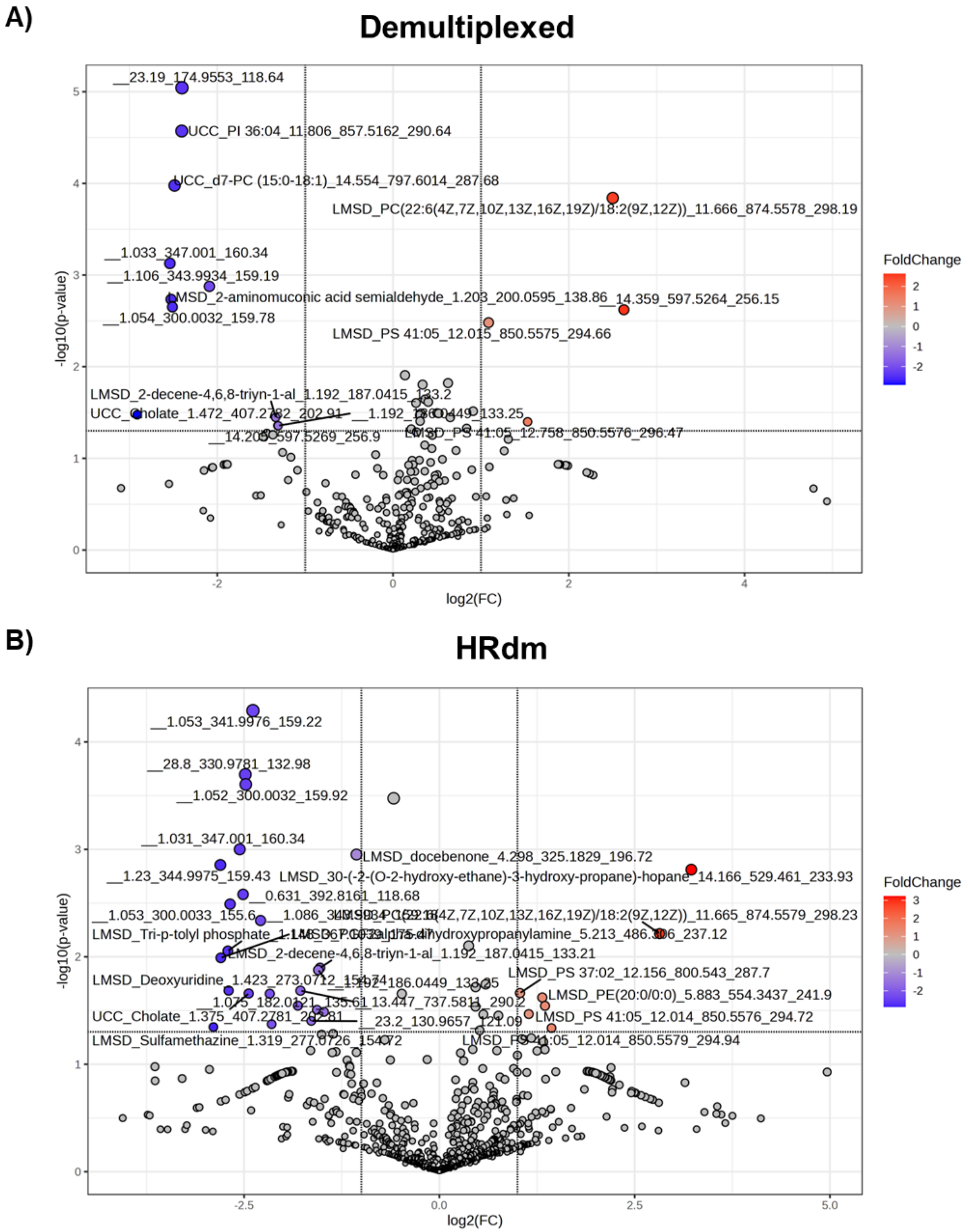


**Figure S2.** Volcano plots from MetaboAnalyst 6.0 between functional and dysfunctional ATP10D mouse models for demultiplexed **(A)** and HRdm **(B)** acquisition modes in negative mode with annotations including the identification database, lipid identification, retention time, mass-to-charge, and CCS value denoted above the features with a p value < 0.05 and a Log2 fold change > 2.

**Table S8.** PC 36:5 ion signals in murine models for demultiplexed and HRdm in positive mode.

| | | | | | Ion Signal | | | | | |
|---|---|---|---|---|---|---|---|---|---|---|
| | | | | | Functional ATP10D | | | Dysfunctional ATP10D | | |
| Acquisition | RT | m/z | CCS | | Mouse 1 | Mouse 2 | Mouse 3 | Mouse 4 | Mouse 5 | Mouse 6 |
| Demultiplexed | 12.31 | 782.5695 | 285.59 | | 2450 | 2709 | 2459 | 5577 | 4583 | 3721 |
| | | | | | | | | | | |
| HRdm (Peak 1) | 12.31 | 782.5695 | 285.39 | | 6373 | 7521 | 8043 | 19798 | 15030 | 12765 |
| HRdm (Peak 2) | 12.31 | 782.5695 | 287.78 | | 0.001 | 0.001 | 0.001 | 5773 | 4996 | 1903 |
